# Water and Energy Turnover in Chinese Young Adults: A Doubly Labeled Water Study of Metabolic Coupling

**DOI:** 10.3390/nu18081268

**Published:** 2026-04-17

**Authors:** Xing Wang, Chang Qu, Jianfen Zhang, Na Zhang

**Affiliations:** 1 Department of Nutrition and Food Hygiene, School of Public Health, Peking University, 38 Xue Yuan Road, Haidian District, Beijing 100191, China; wangxing_98@126.com (X.W.); quchang@ninh.chinacdc.cn (C.Q.); 2 Clinical Nutrition Department, Ningbo Medical Center Li Huili Hospital, 1111 Jiangnan Road, Yinzhou District, Ningbo 315000, China; 3 Office of National Nutrition Plan, National Institute for Nutrition and Health, Chinese Center for Disease Control and Prevention, 27 Nanwei Road, Xicheng District, Beijing 100050, China; 4 Department of Student Nutrition, National Institute for Nutrition and Health, Chinese Center for Disease Control and Prevention, 27 Nanwei Road, Xicheng District, Beijing 100050, China; 5 Laboratory of Toxicological Research and Risk Assessment for Food Safety, Peking University, 38 Xue Yuan Road, Haidian District, Beijing 100191, China

**Keywords:** doubly labeled water, water turnover, energy expenditure, young adults

## Abstract

Background: Accurate estimation of water and energy requirements is fundamental for establishing dietary reference values in young adults. However, evidence integrating objectively measured energy expenditure with detailed water turnover components remains limited in Chinese populations. Objectives: To quantify water intake, water loss, and energy expenditure in healthy young college students, and to examine how energy metabolism is associated with specific components of water turnover under free-living conditions. Methods: Twenty-one healthy adults aged 18–25 years participated in a 14-day observational study conducted in Beijing, China. Total energy expenditure (TEE) was measured over 14 days using the doubly labeled water (DLW) method. Physical activity was monitored over 7 consecutive days using a triaxial accelerometer. Water intake was assessed using multiple methods: water from beverages (including plain drinking water and other beverages) was recorded over 7 days using 24 h fluid intake records, while water from food was measured during days 5–7 using weighed food records combined with duplicate portion and direct drying methods. Urinary and fecal water loss were quantified using 24 h collections conducted during days 5–7. Metabolic water production and insensible water losses were estimated using established physiological equations. Multivariable linear regression analyses were conducted to examine associations between energy-related variables and components of water turnover. Results: Mean total daily water intake was 3023 mL, with water from beverages accounting for 54.1%, water from food for 36.7%, and metabolic water for 9.1%. Mean total daily water loss was 1931 mL, predominantly from urinary excretion (81.0%). DLW-measured TEE averaged 2018.6 kcal/day and was higher in males than in females. Most regression models examining total water intake and beverage-derived water were not statistically significant, and no consistent associations were observed between these variables and total energy intake, TEE, or PAEE. In contrast, TEE was positively associated with metabolic water production and respiratory water loss (both *p* < 0.001). Significant associations with total energy intake were observed for water from food and fecal water loss (both *p* < 0.01), whereas other water intake components showed no significant associations. Conclusions: In young adults, energy metabolism appears to be more closely associated with physiologically regulated components of water turnover than with voluntary water intake. These findings suggest a divergence between endogenous and behaviorally regulated pathways of water turnover and highlight the importance of considering component-specific water dynamics when examining hydration and energy balance, although confirmation in larger studies is warranted.

## 1. Introduction

Water and energy balance are fundamental to human physiology and play essential roles in maintaining metabolic function, thermoregulation, and overall health. Water accounts for approximately 60–70% of body weight in healthy adults and is involved in nutrient transport, waste elimination, cellular homeostasis, and numerous biochemical reactions [1,2]. Both insufficient and excessive water intake have been associated with adverse health outcomes, including impaired cognitive performance, reduced physical capacity, electrolyte imbalance, and increased disease risk [3,4,5,6,7]. Establishing accurate reference values and physiologically grounded reference values for water intake therefore remains an important public health priority, particularly for young adults whose hydration behaviors and metabolic demands are highly variable.

Daily water requirements are met through three principal sources: water from beverages (including plain drinking water and other beverages), water contained in foods, and metabolic water generated during the oxidation of macronutrients [8]. Water loss occurs through urine, feces, respiration, and skin evaporation [9]. Importantly, water intake and water turnover are not equivalent concepts. While water intake reflects behaviorally regulated processes influenced by habits, preferences, and environmental factors, water turnover encompasses both intake and physiologically regulated losses and endogenous production. In addition, different sources of water intake may exert distinct physiological and behavioral effects. For example, plain drinking water and caloric beverages may differentially influence energy intake and metabolism, highlighting the importance of distinguishing between water sources [10,11,12]. However, precise quantification of these components under free-living conditions remains challenging. Conventional dietary assessment methods often underestimate both beverage and food water intake, while non-renal water losses are difficult to measure accurately outside laboratory settings [13,14]. These limitations contribute to uncertainty in estimating true water requirements, particularly in young adult populations.

From a physiological perspective, water turnover is closely linked to energy metabolism. Metabolic water is produced directly through substrate oxidation [15], and higher energy expenditure is associated with increased respiratory ventilation and carbon dioxide production, thereby elevating respiratory water loss [16]. Digestive activity associated with higher energy intake may also influence fecal water loss, as both fecal output and stool water content are influenced by dietary intake [13,17]. In addition to these physiological links, emerging evidence suggests that water intake may influence both energy intake and energy expenditure. Increased consumption of plain drinking water has been associated with lower total energy intake, partly through displacement of caloric beverages and incomplete energy compensation [12], whereas caloric beverages may contribute to higher energy intake due to weaker satiety responses [10]. Furthermore, hydration status and changes in plasma osmolality have been implicated in metabolic regulation, with potential effects on thermogenesis and energy expenditure [18]. Despite these findings, most previous studies have examined the relationships between water intake and energy intake or energy expenditure separately. Empirical evidence integrating both aspects within a unified framework—particularly one that distinguishes between voluntary water intake and physiologically regulated components of water turnover under free-living conditions—remains limited, highlighting the need for studies that simultaneously quantify water turnover components and energy metabolism under real-world conditions.

The doubly labeled water (DLW) method is recognized as the gold standard for assessing total energy expenditure (TEE) under habitual living conditions [19], and uniquely enables the integration of energy metabolism with water turnover processes through isotope kinetics. However, DLW-based studies in Chinese populations, especially among young college students, remain limited. Moreover, research integrating DLW with detailed quantification of water intake, water loss pathways, dietary composition, and physical activity is scarce [20,21]. This evidence gap is particularly relevant for China, where dietary patterns, food moisture content, body composition, and lifestyle behaviors differ from those of Western populations on which many existing reference values are based [22,23].

To address these gaps, the present study combined the DLW method with high-accuracy assessment of water intake, complete collection of urinary and fecal water loss, objective physical activity monitoring, and detailed dietary analysis. The primary aim was to quantify water intake, water loss, and energy expenditure in healthy young college students in Beijing, and to examine how different components of water intake and water turnover are associated with energy metabolism. We hypothesized that energy metabolism would be differentially associated with specific components of water turnover, with stronger relationships expected for physiologically regulated components (e.g., metabolic and respiratory water) than for behaviorally regulated water intake. Additionally, we explored whether different sources of water intake (e.g., plain drinking water vs. other beverages and food-derived water) exhibit distinct associations with energy intake and expenditure. The findings are intended to contribute to a more integrated understanding of the relationships between water turnover and energy metabolism in young adults and may provide evidence to inform future research and potential refinement of water intake and energy requirement recommendations.

## 2. Materials and Methods

### 2.1. Study Design

This study was a 14-day observational investigation conducted under free-living conditions, designed to quantify total water intake, total water loss, and TEE among 21 healthy young adults. The DLW method was used to measure TEE and substrate oxidation. A comprehensive set of assessments was employed to quantify individual components of water turnover, including water from beverages, water from food, metabolic water production, urinary and fecal water loss, as well as respiratory and skin insensible water losses. Physical activity and microenvironmental conditions were also monitored.

**Figure 1:**
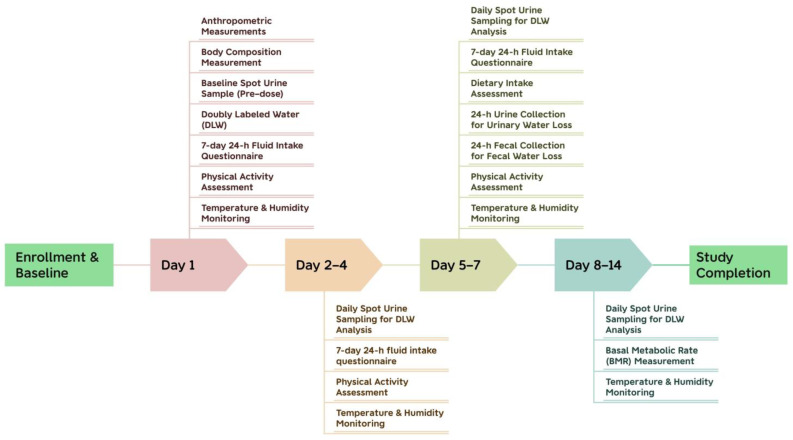
Study Procedure.

Baseline urine samples and DLW dosing were conducted on Day 1. Water from beverages was recorded for 7 consecutive days (Days 1–7), and duplicate-portion food sampling and biological sample collection were conducted on Days 5–7. Physical activity was monitored continuously for 7 days. Basal metabolic rate was measured after completion of the DLW period (Days 8–14), while environmental monitoring continued throughout the study. A study flowchart is provided in Figure 1.

The study was conducted in Beijing, China. Participants were instructed to maintain their usual daily routines, including diet, physical activity, and living environment, throughout the study period. Prior to data collection, all participants received standardized training on study procedures, including dietary recording, fluid intake recording, use of measuring tools, and collection of urine and fecal samples. During the study period, investigators maintained regular contact with participants via phone or social media to monitor compliance, provide reminders for sample collection, and address any issues arising during data collection.

### 2.2. Ethical Approval

The study protocol was approved by the Institutional Review Board of Peking University Health Science Center (IRB00001052-22125). All procedures followed the Declaration of Helsinki. Written informed consent was obtained from all participants.

### 2.3. Participants

A total of 21 healthy college students (10 males and 11 females), aged 18–25 years, were recruited from a university in Beijing in April 2023. Eligibility criteria included:

(1) BMI 18.5–23.9 kg/m^2^;
(2) stable body weight for ≥3 months;
(3) absence of acute or chronic diseases;
(4) no smoking, alcohol abuse, or regular medication use;
(5) low-to-moderate habitual physical activity.

Participants with fever, gastrointestinal symptoms, or medical conditions affecting water or energy metabolism were excluded.

Daily body weight was measured every morning during Days 1–7 to confirm weight stability during DLW measurement.

### 2.4. Sample Size Calculation

The sample size was calculated using the following formula:

$$n={\left(\frac{Z_{\alpha /2}CV}{\varepsilon }\right)}^{2}$$

where *α* = 0.05, corresponding to a *Z*-value of 1.96. The maximum relative error *ε* was set at 10%, and the coefficient of variation was set to 0.15, based on previous research [24]. To account for potential dropout or non-compliance, 10% was added to the calculated sample size. After rounding, the minimum number of participants required was 10 males and 10 females, for a total of 20 participants. This sample size aligns with previous studies in China using the doubly labeled water method, which typically involved 20 participants [13,20]. Based on these considerations, a final sample size of 20 participants was determined.

### 2.5. Anthropometry and Body Composition

Height and weight were measured using a calibrated stadiometer (precision: 0.1 cm) and a digital scale (precision: 0.1 kg) (HDM-300; Huaju Co., Yiwu, China). Self-reported values were collected for comparison but not used in the analysis. Body composition (fat mass, fat-free mass, total body water, extracellular water, intracellular water) was assessed using a bioelectrical impedance analyzer (InBody 770; InBody Co., Seoul, Republic of Korea) under standardized conditions [25].

### 2.6. Microenvironment Assessment

Ambient temperature and relative humidity in participants’ daily living environments were measured using a uniform thermohygrometer (Model 8813; Deli Co., Ningbo, China). Trained investigators recorded measurements at 10:00, 14:00, and 20:00 each day at four locations: sports ground, classrooms, male dormitories, and female dormitories. Absolute humidity was incorporated into the equations for respiratory and insensible skin water loss (Section 2.9.3 and Section 2.9.4).

### 2.7. Doubly Labeled Water Procedure

#### 2.7.1. DLW Dosing and Urine Sampling

TEE was measured using the DLW method. On Day 1, a baseline urine sample was collected at 06:00 prior to dosing, and fasting body weight was measured. Each participant then received a premixed dose of DLW (^2^H_2_O and H_2_^18^O; 1.0 g/kg body weight) prepared according to International Atomic Energy Agency (IAEA) recommendations. The isotopic composition of the dose was 7.96 atom% for ^2^H and 9.81 atom% for ^18^O. Doses were weighed to the nearest 0.1 mg using an analytical balance and administered under investigator supervision.

Participants ingested the DLW dose at 08:00, followed by rinsing the bottle twice with distilled water to ensure complete dose consumption. On the dosing day, spot urine samples (2 mL) were collected at 2, 4, 6, and 8 h post-dose (10:00, 12:00, 14:00, and 16:00), with duplicate aliquots obtained at each time point to characterize isotopic equilibration.

From Day 2 to Day 14, a daily spot urine sample (2 mL, in duplicate) was collected each morning at approximately 07:30. All urine samples were stored at −40 °C until analysis. Isotope enrichments of ^2^H and ^18^O were determined using isotope ratio mass spectrometry, and TEE was calculated using standard DLW equations.

#### 2.7.2. DLW Calculations

The DLW analysis was performed according to the IAEA-recommended two-pool model [26]. In this model, deuterium and oxygen-18 are assumed to distribute within the body water pool and are eliminated at different rates, reflecting water turnover and carbon dioxide production, respectively. The difference between the elimination rate constants of oxygen-18 (*k_O_*) and deuterium (*k_D_*) was used to estimate carbon dioxide production (*rCO*_2_). Isotope dilution spaces for deuterium (*N_D_*) and oxygen-18 (*N_O_*) were calculated from post-dose isotope enrichments, and total body water (TBW) was derived after applying standard correction factors. The elimination rate constants for deuterium (*k_D_*) and oxygen-18 (*k_O_*) were determined from the log-linear disappearance of isotope enrichment over time.

Carbon dioxide production rate (*rCO*_2_) was calculated using the IAEA-recommended equation:

*rCO*_2_ (mol) = 0.455 × *TBW* (mol) × (1.007 × *k_O_* − 1.041 × *k_D_*).

TEE was subsequently derived using the modified Weir equation. The food quotient (FQ), calculated from dietary macronutrient composition, was used as a proxy for the respiratory quotient (RQ) [27,28], and was calculated as:

*FQ* = *VCO*_2_ (L/day)/*VO*_2_ (L/day).

where *VCO*_2_ represents carbon dioxide production and *VO*_2_ represents oxygen consumption, estimated from macronutrient intake using standard stoichiometric equations:

*VO*_2_ (L/day) = 0.966 × *protein intake* (g) + 2.019 × *fat intake* (g) + 0.829 × *carbohydrate intake* (g)

*VCO*_2_ (L/day) = 0.774 × *protein intake* (g) + 1.427 × *fat intake* (g) + 0.829 × *carbohydrate intake* (g)

TEE was calculated as:

*TEE* (kcal/day) = 22.414 × *rCO*_2_ (mol) × (1.10 + 3.90/*RQ*).

Metabolic water production was estimated based on DLW-derived macronutrient oxidation rates using established stoichiometric coefficients. All isotope enrichment measurements were performed in duplicate.

### 2.8. Assessment of Water Intake

#### 2.8.1. Water from Beverages

Water from beverages, including plain water, tea, coffee, milk, and sugar-sweetened beverages, was assessed using a validated 7-day, 24 h fluid intake questionnaire [29]. Participants recorded all drinking events using standardized, calibrated, graduated drinking cups provided by the study team, documenting all beverage types. Fluid volumes were converted to mass assuming a density of 1.0 g/mL. For subsequent analyses, water from beverages was further categorized into plain drinking water and other beverage sources.

#### 2.8.2. Water from Food

Water intake from food was assessed during Days 5–7 using the weighing method combined with the duplicate portion method. Participants prepared an identical portion of all foods consumed. Duplicate samples were weighed, homogenized, and analyzed for moisture content using a direct oven-drying method at 105 °C to constant weight, in accordance with the Chinese National Standard for food moisture determination (GB 5009.3–2016) [30].

#### 2.8.3. Metabolic Water

Metabolic water production was estimated based on macronutrient oxidation-derived TEE measured by the DLW method (Section 2.7), assuming energy balance under free-living conditions. The relative contributions of fat, protein, carbohydrate, and alcohol oxidation were approximated using their respective proportions in total energy intake.

Metabolic water production was calculated using the following equation [31]:

*Metabolic water* (L/day) = *TEE* (kcal/day) × [(%*fat* × 0.119) + (%*protein* × 0.103) + (%*carbohydrate* × 0.150) + (%*alcohol* × 0.168)]/1000

where the coefficients represent the amount of water (g) produced per kcal of energy derived from each macronutrient, and division by 1000 converts grams to liters.

Total water intake was defined as the sum of water from beverages, water from food, and metabolic water. Water from beverages was assessed as the 7-day mean (Days 1–7), while water from food was measured over 3 days (Days 5–7) and assumed to be representative of habitual intake.

### 2.9. Assessment of Water Loss

#### 2.9.1. Urinary Water Loss

All urine voided by participants during Days 5–7 was collected continuously over 24 h for three consecutive days using standardized urine collection bags. Total urine output was weighed after each voiding event, and daily urinary water loss was calculated accordingly.

#### 2.9.2. Fecal Water Loss

All feces excreted during Days 5–7 were collected continuously over 24 h for three consecutive days. Total fecal output was weighed after each defecation, and approximately 2 g of each sample was collected and stored at −40 °C for analysis. Fecal water content was determined using the same direct drying method described above, and total fecal water loss was calculated as fecal weight multiplied by fecal water content [30].

#### 2.9.3. Respiratory Water Loss

Respiratory water loss was estimated based on ventilation volume and ambient absolute humidity. Ventilation (L/day) was derived from carbon dioxide production (rCO_2_) measured by the DLW method. Following previously published approaches, a constant fractional concentration of CO_2_ in expired air of 3.5% was assumed to estimate ventilation under free-living conditions [31]. Respiratory water loss was calculated as [32]:

*Respiratory water loss* (L/day) = *ventilation* (L/day) × *absolute humidity* (mg/L)/1000.

where absolute humidity was derived from measured relative humidity and ambient temperature under standard atmospheric pressure conditions.

#### 2.9.4. Skin Water Loss

Skin insensible water loss was estimated using ambient absolute humidity and body surface area according to established equations [31,33]. The calculation was as follows:

*Body surface area* (m^2^) = [*height* (m)] ^0.725^ × [*weight* (kg)] ^0.425^ × 0.007184

*Skin water loss* (L/day) = 0.18 × [*absolute humidity* (mg/L)/21.7] × *body surface area* (m^2^) × 1.44

Total water loss was calculated as the sum of urinary, fecal, respiratory, and skin water loss.

### 2.10. Energy Intake Assessment

Energy intake was assessed concurrently with duplicate-portion food sampling. Moisture-free food samples were analyzed for protein, fat, and carbohydrate content. Energy was calculated using Atwater factors (protein 4 kcal/g, carbohydrate 4 kcal/g, fat 9 kcal/g).

### 2.11. Physical Activity Measurement

Physical activity energy expenditure (PAEE) was assessed using a triaxial accelerometer (ActiGraph wGT3X-BT, ActiGraph LLC, Pensacola, FL, USA), worn on the right hip for 7 consecutive days. The ActiGraph accelerometer has been widely validated for the assessment of physical activity and estimation of energy expenditure in both laboratory and free-living conditions, showing good agreement with indirect calorimetry and doubly labeled water methods [34,35,36]. A valid day was defined as at least 10 h of wear time. Participants were included in the analysis if they had at least two valid weekdays and one valid weekend day of accelerometer data, to ensure representation of habitual physical activity patterns. Data were collected at a sampling rate of 30 Hz and processed into counts using standard ActiGraph procedures. Activity intensity was classified into sedentary, light, and moderate-to-vigorous physical activity based on established cut-points [37]. PAEE was estimated from accelerometer-derived activity counts using validated prediction equations and represents activity-related energy expenditure above resting levels.

### 2.12. Basal Metabolic Rate

Basal metabolic rate (BMR) was measured using a portable indirect calorimetry system (MetaMax 3B; Cortex, Leipzig, Germany) by trained investigators following standardized protocols [38,39]. Participants fasted overnight, avoided caffeine and vigorous activity for 24 h, and rested quietly in a supine position. Measurements were performed using a ventilated face mask connected to the device, with participants breathing calmly for 20–30 min until gas exchange data stabilized. Parameters including BMR, oxygen consumption, carbon dioxide production, and respiratory quotient were recorded and exported for analysis. Measured BMR values were used as basal energy expenditure (BEE) in subsequent calculations of total energy requirements.

### 2.13. Quality Control

All investigators (*n* = 10) received standardized training on study procedures, including questionnaire administration, dietary assessment, accelerometer use, and collection of urine and fecal samples. Standardized and calibrated instruments (including digital scales, temperature–humidity meters, and electronic balances) were used throughout the study to ensure consistency of measurements. All procedures were routinely checked to maintain data quality. For dietary and fluid intake assessment, each participant was provided with standardized and calibrated weighing tools and graduated containers, along with detailed instructions to ensure accurate and consistent recording. Body weight was measured using standardized digital scales under consistent conditions, with participants instructed to measure body weight in the morning after voiding. Urine sample collection was monitored with regular reminders via social media, and all samples were protected from light and promptly transported to the laboratory for storage and analysis. DLW samples, supplied by Jiangsu Huayi Technology Co., Ltd., Changshu, China (±0.5% accuracy, cGMP-compliant), were analyzed using continuous-flow isotope ratio mass spectrometry. All isotope measurements were performed in duplicate, with reanalysis conducted when analytical precision exceeded 0.1%, and quality control samples were included at regular intervals. All questionnaire data were double-entered and cross-checked by trained personnel to ensure data accuracy.

### 2.14. Statistical Analysis

Statistical analyses were performed using SPSS Statistics version 26.0 (IBM Corp., Armonk, NY, USA). Data were entered using Microsoft Excel 2013 with double data entry to ensure data accuracy.

Normality of continuous variables was assessed using Q–Q plots and the Shapiro–Wilk test. Normally distributed data are presented as mean ± standard deviation (SD) and were compared between groups using independent-sample *t* tests, whereas non-normally distributed data are presented as median (interquartile range) and were compared using the Mann–Whitney *U* test.

Multivariable linear regression analyses were conducted to examine the associations between energy metabolism indicators and components of water intake and water loss under free-living conditions. Variables with skewed distributions (including water from beverages, water from other beverages, water from food, urinary water loss, and fecal water loss) were log-transformed prior to analysis. In these models, individual components of water intake and water loss were treated as dependent variables and analyzed in separate regression models. Independent variables included total energy intake, TEE, PAEE, and body fat percentage, with sex included as a covariate in all models. Given the relatively small sample size, the number of predictors was restricted to key physiological variables to minimize overfitting and ensure model stability. Multicollinearity was assessed using variance inflation factors (VIFs), and no evidence of problematic collinearity was observed. Standardized regression coefficients (*β*) are reported.

All statistical tests were two-sided, and a *p* value of <0.05 was considered statistically significant.

## 3. Results

### 3.1. Participant Characteristics

All 21 participants (10 males and 11 females) completed the 14-day study. Baseline anthropometric and physiological characteristics are presented in Table 1. Male participants were significantly taller and heavier than females and had larger waist circumference, higher systolic blood pressure, lower body fat percentage, and higher proportions of total, extracellular, and intracellular body water relative to body weight (all *p* < 0.05). No significant sex differences were observed in age and body mass index (*p* > 0.05).

### 3.2. Microenvironment Conditions

The study was conducted during the temperate spring season. Over the 14-day survey period, the overall mean ambient temperature was 24.3 °C, with a mean relative humidity (RH) of 28%. Outdoor conditions at the playground were cooler and slightly more humid (21.5 °C and 30% RH), whereas indoor environments—including classrooms and male and female dormitories—were characterized by higher temperatures and similar relative humidity levels (25.2 °C and 28% RH) (Table 2).

Based on the relationship between relative and absolute humidity at 1 bar, the corresponding absolute humidity under the experimental conditions (25 °C, 30% RH) was calculated as 7.58 g/m^3^. This value was used for the estimation of cutaneous and respiratory water loss.

### 3.3. Dietary Energy Intake

Daily dietary energy intake and macronutrient composition were assessed for all participants on Days 5–7. The 3-day mean total energy intake was 2092.3 ± 592.3 kcal/day, with males consuming significantly more energy than females (2331.9 ± 539.0 vs. 1874.3 ± 559.9 kcal/day, *p* = 0.002).

The 3-day mean daily protein intake was 84.4 ± 29.5 g, contributing 337.8 ± 118.1 kcal/day and accounting for 16.1 ± 3.5% of total energy intake. Protein intake and its energy contribution were significantly higher in males than in females (both *p* < 0.05). Mean fat intake was 94.1 ± 32.6 g/day, providing 846.8 ± 293.0 kcal/day (40.1 ± 6.1% of total energy). Fat intake in grams was significantly higher in males than in females (*p* = 0.021), while the energy proportion did not differ significantly by sex (*p* > 0.05). Carbohydrate intake averaged 227.0 ± 66.7 g/day, contributing 907.8 ± 266.9 kcal/day (43.8 ± 7.2% of total energy); males consumed a greater absolute amount of carbohydrates than females (*p* = 0.008), whereas the energy contribution did not differ significantly by sex.

Detailed energy and macronutrient intake data are presented in Table 3.

### 3.4. Daily Energy Expenditure

TEE, measured using the 14-day doubly labeled water method, averaged 2018.6 ± 250.7 kcal/day. Males had significantly higher TEE than females (2211.4 ± 135.1 vs. 1843.3 ± 195.8 kcal/day, *p* < 0.001). BEE averaged 1357.0 ± 164.6 kcal/day and was also higher in males than in females (1462.5 ± 165.7 vs. 1261.1 ± 90.8 kcal/day, *p* = 0.002). Based on accelerometer measurements, participants spent most of the day in sedentary or light-intensity activities. The 7-day mean PAEE was 293.8 ± 94.1 kcal/day, with no significant sex difference (*p* > 0.05). Accelerometer wear time and mean METs did not differ significantly between males and females (Table 4).

### 3.5. Daily Water Sources

Unless otherwise specified, water from beverages represents the 7-day mean (Days 1–7), while water from food represents the 3-day mean (Days 5–7).

The 7-day mean total daily water intake (Days 1–7), comprising plain drinking water, water from other beverages, water from food, and metabolic water, averaged 3023 ± 728 mL among the 21 participants, with no significant difference between males and females (*p* > 0.05). When expressed relative to body weight, total water intake averaged 51.5 ± 12.6 mL/kg/day, with a trend toward higher intake in females than males (55.9 ± 11.9 vs. 46.7 ± 12.1 mL/kg/day, *p* = 0.094). When normalized to TEE, total water intake averaged 1.5 ± 0.4 mL/kcal/day, reflecting the volume of water consumed per unit of energy expended by the body. This value was significantly higher in females than in males (1.7 ± 0.4 vs. 1.3 ± 0.3 mL/kcal/day, *p* = 0.040).

Water from beverages was the predominant source, with a median intake of 1530 mL, accounting for 54.1% of total daily water intake. When disaggregated, plain drinking water contributed a median of 1017 mL/day (38.2% of total water intake), while water from other beverages contributed 309 mL/day (15.1%). Although water from beverages did not differ significantly between sexes, females had a higher proportional contribution than males (58.8 ± 8.6% vs. 49.0 ± 9.1%, *p* = 0.020). No significant sex differences were observed for plain drinking water or water from other beverages individually (all *p* > 0.05).

Water intake from food contributed a median of 1014 mL/day, representing 36.7 ± 9.5% of total water intake, with no significant sex differences in either absolute intake or proportional contribution (*p* > 0.05).

Metabolic water production averaged 262 ± 33 mL/day, accounting for approximately 9.1 ± 2.3% of total daily water intake. Both the absolute amount and proportional contribution of metabolic water were significantly higher in males than in females (both *p* < 0.05). Detailed data are presented in Table 5.

### 3.6. Daily Water Loss

The 3-day mean total daily water loss (Days 5–7), calculated as the sum of urinary, fecal, skin, and respiratory losses, averaged 1931 ± 504 mL among the 21 participants, with no significant difference between males and females (*p* > 0.05). When normalized to body weight, total water loss averaged 32.9 ± 8.7 mL/kg/d, and when expressed per unit of TEE, it averaged 1.0 ± 0.3 mL/kcal/d, with no significant sex differences for either measure (both *p* > 0.05). Urinary water loss was the predominant route of total water loss, with a median volume of 1634 mL, accounting for approximately 81% of total daily water loss. Neither urinary volume nor its proportional contribution differed significantly between sexes (*p* > 0.05). Fecal water loss was minimal, with a median of 76 mL per day (4.8% of total water loss), and showed no significant sex difference. Skin and respiratory water losses contributed comparable proportions to total water loss, averaging 8.3 ± 2.2% and 8.4 ± 2.1%, respectively. Although both skin and respiratory water loss volumes were significantly higher in males than in females (both *p* < 0.001), their relative contributions to total water loss did not differ significantly between sexes. Detailed data are presented in Table 6.

### 3.7. Associations Between Energy Metabolism and Water Turnover Components

Multivariable linear regression analyses were performed to evaluate the associations between energy-related variables and components of water intake and water loss, with sex included as a covariate in all models (Table 7). Non-normally distributed variables, including water from beverages, water from other beverages, water from food, urinary water loss, and fecal water loss, were log-transformed prior to analysis. Model fit statistics (*F* values and corresponding *p* values) are presented to indicate the overall significance of each regression model.

For water intake variables, most models were not statistically significant, including total water intake, water from beverages, plain drinking water, and water from other beverages (all *p* for model > 0.05). No consistent associations were observed between these variables and total energy intake, TEE, PAEE, or body fat percentage. In contrast, the model for water from food was statistically significant (*F* = 4.119, *p* = 0.015). In this model, water from food was positively associated with total energy intake (*β* = 0.855, *p* = 0.001), while no significant associations were observed for TEE, PAEE, or body fat percentage. The model for metabolic water production was highly significant (*F* = 267.316, *p* < 0.001), with TEE showing a strong positive association with metabolic water production (*β* = 1.030, *p* < 0.001). No significant associations were observed for other predictors.

For water loss variables, the models for total water loss and urinary water loss were not statistically significant (both *p* for model > 0.05), and no significant associations were observed. The model for fecal water loss was statistically significant (*F* = 5.613, *p* = 0.005). Total energy intake was positively associated with fecal water loss (*β* = 0.732, *p* = 0.002), whereas other predictors were not significant. The model for skin water loss was also statistically significant (*F* = 8.757, *p* < 0.001); however, none of the independent variables showed statistically significant associations (all *p* > 0.05). The model for respiratory water loss was highly significant (*F* = 198.597, *p* < 0.001), with TEE positively associated with respiratory water loss (*β* = 1.009, *p* < 0.001). No significant associations were observed for total energy intake, PAEE, or body fat percentage.

Overall, energy metabolism showed stronger and more consistent associations with physiologically regulated components of water turnover (metabolic and respiratory water loss) than with voluntary water intake.

## 4. Discussion

This study provides a comprehensive assessment of water intake, water loss, and energy expenditure among healthy young college students in Beijing using the DLW method combined with detailed measurements of drinking behavior, duplicate diet analysis, and complete urine and fecal collections. Overall, no consistent associations were observed between total daily water intake or its voluntary components and indices of energy metabolism, including total energy intake, TEE, and PAEE. Most regression models examining water intake variables were not statistically significant, suggesting that voluntary fluid intake may not directly track metabolic demand under free-living conditions. In contrast, several physiologically regulated components of water turnover showed significant associations with energy metabolism. Metabolic water production and respiratory water loss were positively associated with TEE, while fecal water loss was positively associated with total energy intake. These findings are physiologically plausible and consistent with known mechanisms linking substrate oxidation, carbon dioxide production, and water turnover.

The mean TEE observed in this study (2018.6 kcal/day) is broadly consistent with values reported in previous DLW-based studies of Chinese young adults with similar activity levels [13,40]. This supports the validity of the present measurements and suggests that the study population is comparable to previously studied cohorts.

Previous studies using the DLW method, together with evidence from hydration-focused reviews, indicate that healthy adults living in temperate environments typically consume approximately 2.5–3.5 L of total water per day [5]. At the same time, water turnover can vary widely between individuals due to differences in climate, physical activity, dietary habits, and lifestyle patterns [32]. In the present study, total water intake of Chinese young adults fell within this commonly reported range, suggesting that overall physiological water requirements may be broadly comparable across populations living in temperate climates.

One notable feature of water intake in this cohort was the relatively high contribution of food moisture, which accounted for approximately 37% of total daily water intake. This proportion is higher than the 20–30% typically reported in U.S. and European populations [41,42]. This difference may be explained by dietary characteristics specific to the study population. In particular, typical Chinese dietary patterns, especially in campus dining settings, often include mixed dishes, soups, stews, and vegetable-rich meals, all of which have relatively high water content [43]. While these findings may be representative of certain groups of Chinese young adults, caution is warranted when extrapolating them to populations with different dietary habits.

In this study, total daily water loss was slightly lower than total water intake. This difference may reflect short-term fluctuations in hydration behavior, temporary storage of water within body tissues, and minor methodological mismatches between the assessment windows for intake and loss. The distribution of water loss across different pathways was consistent with established physiological patterns, with urinary water loss accounting for approximately 80% of total water loss under temperate conditions [13].

Sex differences were observed, with males exhibiting significantly higher respiratory and skin water losses than females. This pattern is biologically plausible: males generally have greater body mass, larger surface area, and higher TEE, which together elevate both respiratory and skin insensible water loss. Higher metabolic rates increase CO_2_ production and respiratory evaporation, while a larger skin surface area enhances skin water loss. These findings are consistent with evidence from large multinational datasets indicating that body size, body composition, and energy expenditure are key determinants of water turnover across diverse populations [5,8].

The present study provides a detailed characterization of the relationships between energy metabolism and specific components of water turnover under free-living conditions. A key finding is that energy metabolism was more consistently associated with physiologically regulated components of water turnover than with voluntary water intake.

Specifically, TEE showed positive associations with both metabolic water production and respiratory water loss. These findings are physiologically plausible, as metabolic water is directly generated through substrate oxidation, and increased energy expenditure is accompanied by elevated carbon dioxide production and ventilation, leading to greater respiratory water loss [24,44]. The consistency of these associations supports the internal validity of the DLW-based measurements and highlights the close coupling between energy metabolism and internally regulated water turnover pathways [45].

In contrast, no significant associations were observed between energy-related variables and total water intake or its beverage-related components, including plain drinking water and water from other beverages. This suggests that voluntary drinking behavior may not be tightly regulated by metabolic demand under free-living conditions, and may be influenced by external behavioral and environmental factors, consistent with previous studies [46,47,48]. Recent evidence in young adult populations has further highlighted the role of structured daily routines and lifestyle patterns in shaping hydration-related behaviors [49].

Interestingly, energy intake was positively associated with water from food as well as fecal water loss. This likely reflects the fact that higher food intake contributes both to greater dietary water intake and increased gastrointestinal processing, resulting in higher fecal water excretion [13,50]. These findings indicate that dietary intake plays a more direct role in modulating certain components of water turnover, particularly those related to digestion and absorption.

Taken together, these results suggest a divergence between physiologically driven and behaviorally regulated components of water turnover. Voluntary water intake is primarily regulated by osmoregulatory signals and thirst-driven behavior, rather than directly by energy expenditure, whereas metabolic water production and respiratory water loss are intrinsically linked to substrate oxidation and ventilation [51,52,53].

In addition, the variability structure of these variables may further contribute to the observed findings. Voluntary water intake typically exhibits substantial intra- and inter-individual variability across days due to day-to-day behavioral fluctuations, whereas TEE represents a more stable physiological measure. This discrepancy in variability may attenuate detectable associations, particularly in a relatively small sample. Methodological factors may further contribute to this discrepancy, as the mismatch in measurement periods (14 days for TEE vs. shorter periods for water intake) may reduce the ability to capture synchronized variations between energy expenditure and voluntary water intake.

This distinction may partly explain why total water intake alone may not fully reflect physiological water requirements or metabolic activity. Existing reviews and reference frameworks have also emphasized that water homeostasis is tightly regulated and that adequate water intake values are generally based on observed intakes or hydration-related outcomes rather than a single physiological requirement marker [47,54,55,56,57].

These findings suggest that recommendations based solely on total water intake may overlook important physiological determinants of water turnover linked to energy metabolism. Given the relatively small sample size and the number of regression models performed, the analyses should be considered exploratory and hypothesis-generating. The possibility of type I error due to multiple comparisons cannot be excluded, and therefore, findings with borderline statistical significance should be interpreted with caution.

The strengths of this study include the use of the DLW method, the gold standard for assessing free-living energy expenditure, combined with detailed assessments of water intake and water loss. The study further employed multivariable linear regression models to examine the relationships between energy metabolism and multiple components of water turnover within a unified analytical framework. This approach enabled the simultaneous evaluation of both behaviorally regulated water intake and physiologically regulated water loss pathways, rather than considering these components in isolation. This integrated approach provides a more detailed characterization of the differential relationships between energy metabolism and distinct components of water turnover.

Several limitations should be considered. First, the mismatch in measurement time frames between water intake, water loss, and TEE may limit comparability across variables. Second, methodological and analytical constraints should be acknowledged. The relatively small sample size limited statistical power, particularly in models examining disaggregated components of water intake. In addition, insensible water losses (respiratory and skin) were estimated using established physiological equations rather than directly measured, which required simplifying assumptions (e.g., fixed parameters for expired air composition). Furthermore, some variables included in the regression analyses were derived from shared DLW-based measurements, which may introduce structural dependencies and potential endogeneity, and thereby affect the independence of observed associations, potentially contributing to standardized regression coefficients exceeding unity in multivariable models. Third, certain physiological and contextual factors were not fully captured. Hydration status was not assessed using objective biomarkers, and no data on hormonal regulation were available. In addition, although standardized training and monitoring procedures were implemented, the use of weighed dietary records and repeated biological sample collection under free-living conditions may have influenced participants’ habitual behaviors and introduced measurement error. Incomplete or inaccurate collection of urine and fecal samples cannot be entirely excluded, which may introduce additional variability in the estimation of water loss components. The BMR measurement protocol, which required temporary water restriction and reduced physical activity on a specific study day, may also have introduced short-term behavioral alterations. Finally, the study was conducted within a single season (spring), and seasonal variations in environmental conditions may influence water turnover and energy metabolism [58]. In addition, the relatively homogeneous university setting may have influenced participants’ lifestyle patterns. Structured class schedules, campus-based dining systems, and similar living environments may have reduced variability in dietary intake and PAEE. This may partly explain the relatively limited variation observed in behavioral components and should be considered when generalizing the findings to more diverse populations.

Future research should build upon the limitations of the present study by adopting more comprehensive and aligned study designs. In particular, future studies should aim to assess water intake, water loss, and energy expenditure over consistent time frames to improve comparability of measurements. The inclusion of objective hydration biomarkers (e.g., urine osmolality or specific gravity) would also enable evaluation of the adequacy of water intake in relation to physiological needs. In addition, larger and more diverse populations are needed to improve statistical power and to examine potential heterogeneity in the relationships between different sources of water intake and energy metabolism. Studies including participants with varying dietary patterns, physical activity levels, and environmental exposures would provide more generalizable evidence. Finally, integrating the doubly labeled water method with emerging technologies—such as digital dietary assessment tools, ecological momentary assessment, and real-time monitoring approaches—may further enhance the precision and ecological validity of measurements of water and energy dynamics in free-living conditions.

## 5. Conclusions

In conclusion, this study demonstrates that, among healthy young adults under free-living conditions, energy metabolism is more closely associated with physiologically regulated components of water turnover than with voluntary water intake.

These findings contribute to a more nuanced understanding of the relationships between water turnover and energy metabolism, highlighting a divergence between endogenous and behaviorally regulated pathways of water balance.

The results may help inform future research on the refinement of water Adequate Intake (AI) recommendations, while emphasizing the importance of considering both total and component-specific measures of water intake.

## Figures and Tables

**Table 1:** Baseline Anthropometric and Physiological Characteristics of Participants.

Parameter	Total (*n* = 21)	Male (*n* = 10)	Female (*n* = 11)	*t*	*p*
Age (years)	22.5 ± 1.4	22.2 ± 1.7	22.7 ± 1.1	−0.856	0.403
Height (cm)	166.9 ± 9.1	174.3 ± 5.0	160.1 ± 6.0	5.878	<0.001
Weight (kg)	59.1 ± 6.9	63.5 ± 5.6	55.1 ± 5.5	3.442	0.003
BMI (kg/m^2^)	21.2 ± 1.7	20.9 ± 2.0	21.5 ± 1.4	−0.737	0.470
Body fat (%)	25.0 ± 7.8	19.2 ± 4.8	30.4 ± 5.8	−4.784	<0.001
TBW/BW (%)	54.9 ± 5.8	59.3 ± 3.6	51.0 ± 4.3	4.780	<0.001
ECW/BW (%)	20.8 ± 2.0	22.2 ± 1.5	19.5 ± 1.6	4.002	0.001
ICW/BW (%)	34.1 ± 3.7	37.0 ± 2.1	31.4 ± 2.7	5.218	<0.001
Waist circumference (cm)	74.9 ± 6.3	78.5 ± 5.9	71.7 ± 4.7	2.950	0.008

Note: BMI: Body Mass Index; BW: Body Weight; TBW: Total Body Water; ECW: Extracellular Water; ICW: Intracellular Water. Data are presented as mean ± SD.

**Table 2:** Microenvironment Conditions During the Survey.

Location	Temperature (°C)	RH (%)
Playground (outdoor)	21.5 ± 5.5	30 ± 13
Classroom (indoor)	25.5 ± 0.7	28 ± 5
Female dormitory (indoor)	25.7 ± 0.6	26 ± 8
Male dormitory (indoor)	24.3 ± 1.1	29 ± 7
Mean	24.3 ± 3.3	28 ± 9

Note: RH: Relative Humidity. Data are presented as mean ± SD.

**Table 3:** Daily Energy and Macronutrient Intake of Participants.

Parameter	Total (*n* = 21)	Male (*n* = 10)	Female (*n* = 11)	*t*	*p*
Protein (g)	84.4 ± 29.5	99.2 ± 27.9	71.1 ± 24.4	4.261	<0.001
Protein energy ratio (%)	16.1 ± 3.5	17.1 ± 3.7	15.1 ± 2.9	2.332	0.023
Fat (g)	94.1 ± 32.6	103.9 ± 31.7	85.2 ± 31.1	2.369	0.021
Fat energy ratio (%)	40.1 ± 6.1	39.7 ± 6.0	40.5 ± 6.3	−0.519	0.606
Carbohydrate (g)	227.0 ± 66.7	250.1 ± 63.6	205.9 ± 63.2	2.762	0.008
Carbohydrate energy ratio (%)	43.8 ± 7.2	43.2 ± 7.0	44.4 ± 7.4	−0.636	0.527
Total energy (kcal)	2092.3 ± 592.3	2331.9 ± 539.0	1874.3 ± 559.9	3.301	0.002

Note: Data are presented as mean ± SD. Comparisons between males and females were performed using independent *t*-tests.

**Table 4:** Daily Physical Activity Time and Energy Expenditure.

Parameter	Total (*n* = 21)	Male (*n* = 10)	Female (*n* = 11)	*Z/t*	*p*
TEE (kcal)	2018.6 ± 250.7	2211.4 ± 135.1	1843.3 ± 195.8	4.963	0.001
BEE (kcal)	1357.0 ± 164.6	1462.5 ± 165.7	1261.1 ± 90.8	3.501	0.002
PAEE (kcal)	293.8 ± 94.1	293.9 ± 97.6	293.6 ± 95.6	0.008	0.993
Accelerometer wear time (min)	963.0 (140.0)	958.0 (195.0)	973.5 (141.0)	−1.820	0.069
METs	1.15 (0.10)	1.17 (0.12)	1.15 (0.07)	−1.667	0.096

Note: TEE, total energy expenditure; BEE, basal energy expenditure; PAEE, physical activity energy expenditure. Data are presented as mean ± SD for normally distributed variables (TEE, BEE, and PAEE) and as median (interquartile range) for other parameters. Sex differences were assessed using independent *t*-tests for normally distributed variables and Mann–Whitney *U* tests for non-normally distributed variables.

**Table 5:** Daily Water Intake of Participants.

Parameter	Total (*n* = 21)	Male (*n* = 10)	Female (*n* = 11)	*Z/t*	*p*
Total water intake (mL/day)	3023 ± 728	2959 ± 758	3082 ± 730	−0.378	0.710
Total water intake (mL/kg/day)	51.5 ± 12.6	46.7 ± 12.1	55.9 ± 11.9	−1.761	0.094
Total water intake (mL/kcal/day)	1.5 ± 0.4	1.3 ± 0.3	1.7 ± 0.4	−2.206	0.040
Water from beverages (mL)	1530 (746)	1270 (420)	1600 (1045)	−1.831	0.067
Water from beverages (%)	54.1 ± 9.9	49.0 ± 9.1	58.8 ± 8.6	−2.540	0.020
Plain drinking water (mL/day)	1139 ±458	1158 ±372	1121 ±542	0.180	0.859
Plain drinking water (%)	38.2 ±12.8	39.5 ± 9.8	36.9 ± 15.5	0.461	0.650
Water from other beverages (mL/day)	309 (411)	225 (335)	324 (1040)	−1.690	0.099
Water from other beverages (%)	13.0 (12.0)	9.5 (11.0)	21.9 (24.0)	−1.972	0.051
Water from food (mL/day)	1014 (444)	1022 (736)	991 (413)	−0.845	0.398
Water from food (%)	36.7 ± 9.5	40.8 ± 9.8	33.1 ± 7.9	2.007	0.059
Metabolic water (mL/day)	262 ± 33	287 ± 19	240 ± 26	4.685	<0.001
Metabolic water (%)	9.1 ± 2.3	10.2 ± 2.3	8.2 ± 1.9	2.220	0.039

Note: Data are presented as mean ± SD for normally distributed variables (total water intake, percentage of water from beverages, percentage of plain drinking water, percentage of water from other beverages, percentage of water from food, metabolic water, and percentage of metabolic water) and as median (interquartile range) for non-normally distributed variables (volume of water from beverages, water from other beverages, water from food and percentage of water from other beverages). Sex differences were assessed using independent *t*-tests for normally distributed variables and Mann–Whitney *U* tests for non-normally distributed variables.

**Table 6:** Daily Water Loss of Participants.

Parameter	Total (*n* = 21)	Male (*n* = 10)	Female (*n* = 11)	*Z/t*	*p*
Total water loss (mL/d)	1931 ± 504	1925 ± 435	1937 ± 581	−0.057	0.956
Total water loss (mL/kg/d)	32.9 ± 8.7	30.6 ± 7.9	34.9 ± 9.2	−1.127	0.274
Total water loss (mL/kcal/d)	1.0 ± 0.3	0.9 ± 0.2	1.1 ± 0.3	−1.650	0.115
Urinary water loss (mL/d)	1634 (554)	1528 (558)	1634 (605)	−0.141	0.888
Urinary water loss (%)	81.0 (9.1)	80.6 (9.6)	81.0 (10.8)	−0.282	0.778
Fecal water loss (mL/d)	76 (76)	58 (57)	92 (108)	−1.408	0.159
Fecal water loss (%)	4.8 ± 3.2	4.1 ± 3.3	5.5 ± 3.1	−0.976	0.341
Skin water loss (mL/d)	150 ± 12	160 ± 7	142 ± 9	4.936	<0.001
Skin water loss (%)	8.3 ± 2.2	8.7 ± 2.0	7.9 ± 2.4	0.823	0.421
Respiratory water loss (mL/d)	152 ± 19	167 ± 11	139 ± 15	4.782	<0.001
Respiratory water loss (%)	8.4 ± 2.1	9.0 ± 1.8	7.8 ± 2.3	1.361	0.189

Note: Data are presented as mean ± SD for normally distributed variables (total water loss, fecal water percentage, skin and respiratory water volumes and percentages) and as median (interquartile range) for non-normally distributed variables (urinary water volume and percentage, fecal water volume). Sex differences were tested using the *t*-test for normally distributed variables and the Mann–Whitney *U* test for non-normally distributed variables.

**Table 7:** Associations of Energy-Related Variables with Water Intake and Water Loss.

Measure	Model Fit	Total Energy Intake (kcal)	TEE (kcal)	PAEE (kcal)	Body Fat (%)
Total water intake (mL)	*F* = 1.767, *p* = 0.180	*β* = 0.477, *p* = 0.079	*β* = 0.423, *p* = 0.204	*β* = 0.221, *p* = 0.366	*β* = −0.008, *p* = 0.982
Water from beverages (log mL)	*F* = 1.740, *p* = 0.186	*β* = 0.042, *p* = 0.871	*β* = 0.355, *p* = 0.284	*β* = 0.431, *p* = 0.089	*β* = −0.062, *p* = 0.860
Plain drinking water (mL)	*F* = 0.493, *p* = 0.777	*β* = 0.270, *p* = 0.375	*β* = −0.073, *p* = 0.847	*β* = 0.161, *p* = 0.570	*β* = 0.247, *p* = 0.548
Water from other beverages (log mL)	*F* = 1.435, *p* = 0.268	*β* = −0.180, *p* = 0.503	*β* = 0.493, *p* = 0.157	*β* = 0.341, *p* = 0.185	*β* = −0.283, *p* = 0.440
Water from food (log mL)	*F* = 4.119, *p* = 0.015	*β* = 0.855, *p* = 0.001	*β* = 0.046, *p* = 0.863	*β* = −0.167, *p* = 0.402	*β* = 0.100, *p* = 0.729
Metabolic water (mL)	*F* = 267.316, *p* < 0.001	*β* = −0.015, *p* = 0.671	*β* = 1.030, *p* < 0.001	*β* = −0.011, *p* = 0.740	*β* = −0.080, *p* = 0.101
Total water loss (mL)	*F* = 1.350, *p* = 0.297	*β* = 0.139, *p* = 0.607	*β* = 0.573, *p* = 0.106	*β* = 0.382, *p* = 0.144	*β* = −0.062, *p* = 0.866
Urinary water loss (log mL)	*F* = 1.078, *p* = 0.411	*β* = 0.003, *p* = 0.992	*β* = 0.629, *p* = 0.088	*β* = 0.328, *p* = 0.220	*β* = −0.016, *p* = 0.965
Fecal water loss (log mL)	*F* = 5.613, *p* = 0.005	*β* = 0.732, *p* = 0.002	*β* = −0.356, *p* = 0.228	*β* = 0.163, *p* = 0.375	*β* = 0.129, *p* = 0.609
Skin water loss (mL)	*F* = 8.757, *p* < 0.001	*β* = 0.076, *p* = 0.642	*β* = 0.088, *p* = 0.671	*β* = 0.251, *p* = 0.116	*β* = 0.368, *p* = 0.113
Respiratory water loss (mL)	*F* = 198.597, *p* < 0.001	*β* = 0.003, *p* = 0.944	*β* = 1.009, *p* < 0.001	*β* = −0.022, *p* = 0.549	*β* = −0.056, *p* = 0.306

Note: TEE, total energy expenditure; PAEE, physical activity energy expenditure. Values are standardized regression coefficients (*β*) with corresponding *p* values obtained from multivariable linear regression models. Dependent variables were individual components of water intake and water loss. Independent variables included total energy intake, TEE, PAEE, and body fat percentage. Sex was included as a covariate in all models. Water from beverages, water from other beverages, water from food, urinary water loss, and fecal water loss were log-transformed prior to analysis.

## Data Availability

The data, code book, and analytic code presented in this study are available upon reasonable request from the corresponding author, subject to privacy protections.

## Abbreviations

The following abbreviations are used in this manuscript:

DLW	Doubly labeled water
IAEA	International atomic energy agency
BMI	Body mass index
RH	Relative humidity
TBW	Total body water
BW	Body weight;
ECW	Extracellular water
ICW	Intracellular water
TEE	Total energy expenditure
PAEE	Physical activity energy expenditure
BMR	Basal metabolic rate
BEE	Basal energy expenditure
SD	Standard deviation
